# Interferon-γ/IRF-1 pathway regulatory mechanisms of PD-L1 expression and relevance for immune checkpoint blockade in hepatocellular carcinoma (HCC)

**DOI:** 10.18632/oncotarget.27995

**Published:** 2021-11-09

**Authors:** Yihe Yan, Leting Zheng, Qiang Du, David A. Geller

**Keywords:** hepatocellular carcinoma, HCC, immune checkpoint, PD-L1, IRF-1

The human T cell and NK cell immune system can attack and destroy cancer cells. The immune checkpoints including PD-1, PD-L1, CTLA-4, TIM3, IDO1, LAG3, and BTLA regulating the quantity and function of these effector immune cells limit normal tissue damage, however their abnormal upregulation in the tumor microenvironment (TME) aggravates the exhaustion of immune cells to protect cancer cells from immune surveillance [1]. Among all immune checkpoints, the PD-L1/PD-1 axis has been confirmed as therapeutic target in many malignancies from clinical trials of immune checkpoint blockade (ICB) alone or in combination with other therapeutics. The recent IMbrave150 clinic trial combining atezolizumab with bevacizumab shows encouraging antitumor activity in patients with unresectable hepatocellular carcinoma (HCC) [2]. Combination of PD-L1/PD-1 blockade and local-regional treatment in HCC has also shown improved response rates.

ICB therapy used in HCC and other malignancies has been promising, however, the primary and acquired resistance to ICB therapy limits the clinical values [3]. It is controversial whether PD-L1 expression in HCC can be regarded as predictive biomarker for ICB therapy [4], however, greater expression of PD-L1 in tumor cells predicts significant association with tumor aggressiveness and postoperative recurrence in HCC [5]. Likewise, higher expression of PD-L1 in HCC cells inhibits T cell function in the hepatic TME [6]. Therefore, PD-L1 expression has a key role in the response to ICB therapy. Regulation of PD-L1 expression involve diverse mechanisms including genomic aberrations, inflammatory signaling, oncogenic signaling, microRNA control, and posttranslational modification [7]. Understanding the complex mechanisms regulating PD-L1 expression is important to overcome resistance to PD-L1/PD-1 blockade therapies.

Interferon-γ (IFN- γ) is a key factor in inflammatory signaling pathway regulating PD-L1 expression. IFN-γ produced by tumor-specific T cells or NK cells induces an effective antitumor immune response through enhanced tumor antigen presentation, recruitment of immune cells, and direct pro-apoptotic and anti-proliferative effects on tumor cells. However, continuous IFN- γ exposure can cause expression of PD-L1 by cancer cells, thereby allowing cancer cells to inactivate antitumor T cells [3].

Interferon regulatory factor 1 (IRF-1) is a central transcription factor in the IFN- γ pathway regulating PD-L1 expression. The recent study by Yan et al. shows that IRF-1 antagonizes IRF-2 to upregulate PD-L1 expression in HCC tumor cells through binding to the interferon response element in the PD-L1 promoter [8]. On the other hand, IRF-1 has been shown to activate anti-tumor immunity of T cells, NK and NKT cells through the CXCL10/CXCR3 paracrine axis in murine HCC [9]. Therefore, IRF-1 should be considered a double-edged sword in antitumor immunity. The good side shows that IRF-1 promotes apoptosis and decreases proliferation of tumor cells and activates antitumor host immunity. The bad side is that IRF-1 induces HCC PD-L1 expression which attenuates antitumor immunity through increased exhaustion of effector immune cells. Hence, activating antitumor function and overcoming the suppressive effects of IRF-1 during the host and tumor immune response provides strategies to effectively target HCC.

ICB therapy may combat the adverse effects of IRF-1. Moreover, upregulated IRF-1 expression in cancer cells has the potential to improve ICB therapy response due to increased number of CD8+cytotoxic T cells infiltrating in proximity to PD-L1 positive tumor cells, as well as enrichment and activation of NK and NKT cells [9, 10]. Therefore, developing strategies to increase IRF-1 expression in combination with ICB therapy has clinical merit.

At present, IRF-1 has been identified as predictive biomarker for PD-L1/PD-1 blockade therapy in metastatic melanoma patients [11], since higher IRF-1 level produces an inflammatory microenvironment characterized by enrichment and activation of NK cells and T cells [9]. Meanwhile, the T cell inflamed gene expression profile is a biomarker for ICB therapy [12]. However, whether IRF-1 expression is a useful biomarker to predict ICB response in HCC will require further translational clinical studies.

Besides the IFN-γ/IRF-1 inflammatory pathway, additional oncogenic pathways play an important role in regulating PD-L1 expression in HCC. Furthermore, inflammatory pathways have crosstalk with the oncogenic pathway in regulating PD-L1 and hepatic TME. Our study finds that IFN-γ/IRF-1 can suppress oncogene checkpoint kinase 1 (CHK1) and CHK1 inhibition induces PD-L1 expression in HCC. A promising option is to combine CHK1 inhibition with ICB therapy for HCC.

In summary, IFN-γ/IRF-1 pathway is an important inflammatory pathway regulating PD-L1 expression and antitumor effective immune cells in the hepatic TME. Further exploring the mechanisms of this pathway and crosstalk with oncogenic signaling holds great clinical value in finding new therapeutic targets for HCC and other malignancies.
